# Navigating Treatment Choices for Stress and Urgency Urinary Incontinence Using Graph Theory in Discrete Mathematics

**DOI:** 10.7759/cureus.61315

**Published:** 2024-05-29

**Authors:** Nobuo Okui

**Affiliations:** 1 Dentistry, Kanagawa Dental University, Kanagawa, JPN

**Keywords:** vaginal non-ablation erbium yag laser, transobturator tape, tension-free vaginal tape, graph theory, discrete mathematics, urgency urinary incontinence, stress urinary incontinence

## Abstract

In this study, we propose a method for navigating the choice of treatment for stress urinary incontinence (SUI) and urgency urinary incontinence (UUI) using graph theory in discrete mathematics. Our previous study accumulated data from 150 patients who underwent tension-free vaginal tape (TVT), transobturator tape (TOT), and vaginal non-ablation Erbium YAG laser (VEL) surgeries between 2014 and 2016. Network diagrams were created using this data. The treatments TVT, TOT, and VEL, along with patient characteristics (1-hour pad test: 1-hrPadTest, Overactive Bladder Symptom Score: OABSS), were represented as nodes and edges in the network diagram. We then employed a heuristic function to select the optimal treatment method for the patients with SUI and UUI. This process enables medical professionals to easily navigate the data for patients with both SUI and UUI concerns by calculating the shortest path connecting the 1-hrPadTest and OABSS. These results, which are consistent with those of previous studies, suggest that VEL is the optimal treatment. Unlike previous studies that employed statistical knowledge that is challenging for patients to understand, our study aids patients in visually comprehending and developing a customized treatment plan. This approach introduces a novel perspective for clinical decision-making in the treatment of urinary incontinence. To the best of our knowledge, this is the first study to apply discrete mathematics to patient decision-making for urinary incontinence treatment.

## Introduction

Discrete mathematics is a branch of mathematics that primarily deals with discrete objects [1]. The algorithms used in car navigation systems employ graph theory from discrete mathematics [1]. This graph theory is used in Google Maps and air traffic control maps, but its application in clinical medicine has been minimal [2]. Our research focused on the utilization of discrete mathematics in defining the treatment [3]. In this study, we used network diagrams and heuristic functions, which are fundamental to car navigation, for treatment selection. A network diagram, which is part of graph theory, visualizes how devices in a network are interconnected [4]. A heuristic function estimates the shortest path cost from a node to a goal [5].

The focus of this study was urinary incontinence, which encompasses stress urinary incontinence (SUI), characterized by leakage during coughing or sneezing, and urgency urinary incontinence (UUI), marked by a sudden urge to urinate [6]. Various treatments for SUI have been explored [6]. In our previous research, as a database for this study, we investigated SUI treatments such as tension-free vaginal tape (TVT) and transobturator tape (TOT), which involve inserting polypropylene mesh tape beneath the mid-urethra, as well as vaginal non-ablation Erbium YAG laser treatment (VEL, FotonaSmooth™ XS, Fotona doo, Ljubljana, Slovenia). Although TVT, TOT, and VEL showed similar effectiveness in patients with SUI, the combination of SUI and UUI presented a challenge. Specifically, some patients who underwent TVT reported worsened UUI, whereas VEL has emerged as a promising option [6]. However, conveying these complex results to patients necessitates advanced knowledge, making treatment selection particularly difficult for those suffering from both SUI and UUI [6-8]. Consequently, there is a clear need for a navigational tool to assist patients in making well-informed decisions.

The hypothesis of this study is that it is possible to optimize the selection of urinary incontinence treatments for individual patients based on their symptoms and treatment history, using network diagrams and heuristic functions from discrete mathematics. Through this approach, we aimed to rapidly and accurately recommend the best treatment options for patients.

## Technical report

The goal of this technological approach is to create a visually intuitive discrete mathematical method to support surgical choices. We have previously reported the application of graph theory in discrete mathematics to clinical medicine [3]. In this project, we developed a Python program code that uses a heuristic function based on network diagrams created using graph theory to assist in surgical decision-making. This study focused on choosing between three treatment strategies - TVT, TOT, and VEL - using data from previous research. Our previous study indicated that all three strategies were effective for patients with SUI within a year after treatment. However, for patients with both SUI and UUI, VEL was the only treatment that led to an improvement.

This study was approved by the Ethics Committee of Yokosuka Urogynecology and Urology. The subjects of the data collected in the previous study were women aged between 20 and 65 who visited our clinic between 2014 and 2016 and were diagnosed with SUI [6]. All patients provided informed consent prior to treatment and written consent was obtained. In the previous study, surgical choices were sequentially applied to 50 patients each who underwent TVT surgery in 2014 [6], TOT surgery in 2015 [6], and VEL [6] (abbreviated as Er. YAG in the previous study) in 2016. The ages of the patients were: TVT group, 48.7 ± 13.9 years; TOT group, 47.8 ± 13.9 years; and laser therapy group, 50.3 ± 13.2 years. The VEL setting involved inserting the laser handpiece into the vagina, setting the wavelength to 2,940nm, and irradiating for 20 minutes in “Smooth mode.” Initially, the entire anterior wall of the vagina was irradiated for 10 min, followed by irradiation for 5 min on the entire vagina and 5 min around the urethra. VEL was performed three times at one-month intervals [6].

Discrete mathematical graph theory

In this study, network graphs were used to visualize and analyze the correlations between various variables. For network analysis, we employed the “NetworkX” library in Python (Python Software Foundation, Beaverton, USA).

The nodes in the network diagram are as follows: a: TVT, b: TOT, c: VEL, d: AGE, e: PVR, f: Δ1-hrPadTest, g: ΔOABSS, h: ΔICIQ-SF.

The edges between a: TVT, b: TOT, and c: VEL are not included. Instead, the edges were established based on the correlation coefficients among all other variables. The lengths of these edges were determined using the inverse of the correlation coefficients, ensuring that a higher correlation coefficient corresponded to a shorter edge. The nodes representing a: TVT, b: TOT, and c: VEL are colored orange, whereas all other nodes are colored light blue. d: AGE represents the age at the time of surgery. e: PVR is the post-void residual volume (mL) one year after surgery. f: Δ1-hrPadTest is the improvement in the 1-hour pad test (g) from before surgery to one year after surgery. The 1-hour pad test, an objective measure, involves leakage into the pad during one hour of specified drinking and exercise. g: ΔOABSS is the improvement in the total Overactive Bladder Symptom Score (OABSS) from before surgery to one year after surgery. OABSS is used as a subjective indicator of the UUI. This deterioration resulted in negative scores. h: ΔICIQ-SF is the improvement in the impact of urinary incontinence on life as measured by the ICIQ-SF from before surgery to one year after surgery. It is a subjective indicator that includes both SUI and UUI. Deterioration results in negative scores.

**Figure 1:**
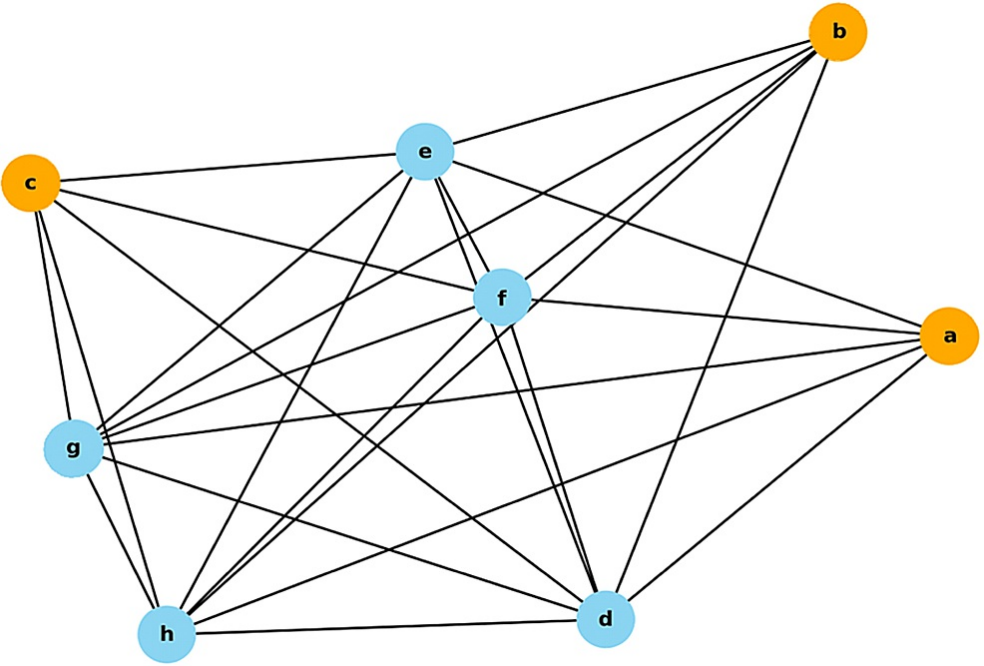
Network diagram with nodes representing three surgical procedures and five variables a: TVT, b: TOT, c: VEL, d: AGE, e: PVR, f: Δ1-hrPadTest, g: ΔOABSS, h: ΔICIQ-SF The nodes for the treatment methods are shown in orange. Other variables are shown in blue. The length of the edges represents the correlation coefficients shown in Table 1. TVT: Tension-free Vaginal Tape, TOT: Transobturator Tape, VEL: Vaginal non-ablation Erbium YAG laser, PVR: Post-void Residual, 1-hrPadTest:1-hour Pad Test, OABSS: Overactive Bladder Symptom Score, ICIQ-SF: International Consultation on Incontinence Questionnaire Short Form. Δ1-hrPadTest: The value obtained by subtracting the post-treatment 1-hrPadTest score from the pre-treatment score, g: ΔOABSS: The value obtained by subtracting the pre-treatment OABSS score from the post-treatment score, h: ΔICIQ-SF: The value obtained by subtracting the pre-treatment ICIQ-SF score from the post-treatment score.

Figure 1 shows the network diagram. Table 1 shows the correlation coefficients that formed the data for the edges. The correlation coefficients used in the network graph were Pearson’s and Spearman's rank correlation coefficients.

**Table 1:** Correlation coefficients between nodes in the network diagram TVT: Tension-free Vaginal Tape, TOT: Transobturator Tape, VEL: Vaginal non-ablation Erbium YAG laser, PVR: Post-void Residual, 1-hrPadTest:1-hour Pad Test, OABSS: Overactive Bladder Symptom Score, ICIQ-SF: International Consultation on Incontinence Questionnaire Short Form. Δ1-hrPadTest: The value obtained by subtracting the post-treatment 1-hrPadTest score from the pre-treatment score, g: ΔOABSS: The value obtained by subtracting the pre-treatment OABSS score from the post-treatment score, h: ΔICIQ-SF: The value obtained by subtracting the pre-treatment ICIQ-SF score from the post-treatment score.

Edges	Correlation coefficients
g: ΔOABSS, c: VEL	0.582454
g: ΔOABSS, b: TOT	0.325493
f: Δ1-hrPadTest, h: ΔICIQ-SF	0.287037
e: PVR, a: TVT	0.274128
g: ΔOABSS, a: TVT	0.242488
e: PVR, c: VEL	0.234225
d: AGE, g: ΔOABSS	0.210861
e: PVR, f: Δ1-hrPadTest	0.170691
d: AGE, f: Δ1-hrPadTest	0.165345
d: AGE, h: ΔICIQ-SF	0.107183
d: AGE, e: PVR	0.104595
h: ΔICIQ-SF, c: VEL	0.090715
f: Δ1-hrPadTest, g: ΔOABSS	0.072268
f: Δ1-hrPadTest, c: VEL	0.071286
g: ΔOABSS, h: ΔΔ	0.068217
e: PVR, g: ΔOABSS	0.063236
h: ΔICIQ-SF, b: TOT	0.049747
f: Δ1-hrPadTest, a: TVT	0.042440
h: ΔICIQ-SF, a: TVT	0.038756
e: PVR, h: ΔICIQ-SF	0.030084
f: Δ1-hrPadTest, b: TOT	0.027618
e: PVR, b: TOT	0.024066
d: AGE, a: TVT	0.018452
d: AGE, b: TOT	0.014776
d: AGE, c: VEL	0.003019

Selective navigation for patients with both SUI and UUI

To facilitate the visual ease of treatment selection for patients with both SUI, indicated by the 1-hrPadTest, and UUI, indicated by the OABSS, a program will be developed to navigate the optimal treatment method among “a: TVT”, “b: TOT”, or “c: VEL”, based on the treatment effects of both variables “f: Δ1-hrPadTest” and “g: ΔOABSS”. In this study, the following approach was adopted.

First, the objective of the navigation program is to search for routes that include “f: Δ1-hrPadTest”, “g: ΔOABSS”, and the three treatment methods. Then, it proposes the method that represents the shortest route, in other words, the method that offers the most comprehensive effect.

Code Example

Below is an example of Python code designed to solve this problem. This code evaluates the impact of each surgical method on “f: Δ1-hrPadTest” and “g: ΔOABSS” and selects the most effective method.

def heuristic_function(correlations, problem1, problem2):

 """

 Estimate the most effective treatment based on the correlation coefficients for the given problems.

:param correlations: List of edges and correlation coefficients

:param problem1: The first problem (e.g., 'f: Δ1-hrPadTest' here)

:param problem2: The second problem (e.g., 'g: ΔOABSS' here)

:return: The most effective treatment

 """

 # Calculate the sum of correlation coefficients for each treatment

 treatment_scores = {treatment: 0 for treatment in ['a: TVT', 'b: TOT', 'c: VEL']}

 for edge in correlations:

 node1, node2, coeff = edge

 if node1 in treatment_scores and (problem1 in node2 or problem2 in node2):

 treatment_scores[node1] += coeff

 elif node2 in treatment_scores and (problem1 in node1 or problem2 in node1):

 treatment_scores[node2] += coeff

 

 # Select the treatment with the highest score

 best_treatment = max(treatment_scores, key=treatment_scores.get)

 return best_treatment

# Create a list of correlation coefficients

correlations = [

 ('g: ΔOABSS', 'c: VEL', 0.582454),

 ('g: ΔOABSS', 'b: TOT', 0.325493),

 ('g: ΔOABSS', 'a: TVT', 0.242488),

 ('f: Δ1-hrPadTest', 'h: ΔICIQ-SF', 0.287037),

 ('e: PVR', 'a: TVT', 0.274128),

 ('e: PVR', 'c: VEL', 0.234225),

 ('f: Δ1-hrPadTest', 'g: ΔOABSS', 0.072268),

 ('f: Δ1-hrPadTest', 'c: VEL', 0.071286),

 ('f: Δ1-hrPadTest', 'a: TVT', 0.042440),

 ('f: Δ1-hrPadTest', 'b: TOT', 0.027618),

]

# Use the heuristic function to find the optimal treatment

best_treatment = heuristic_function(correlations, 'f: Δ1-hrPadTest', 'g: ΔOABSS')

best_treatment

**Figure 2:**
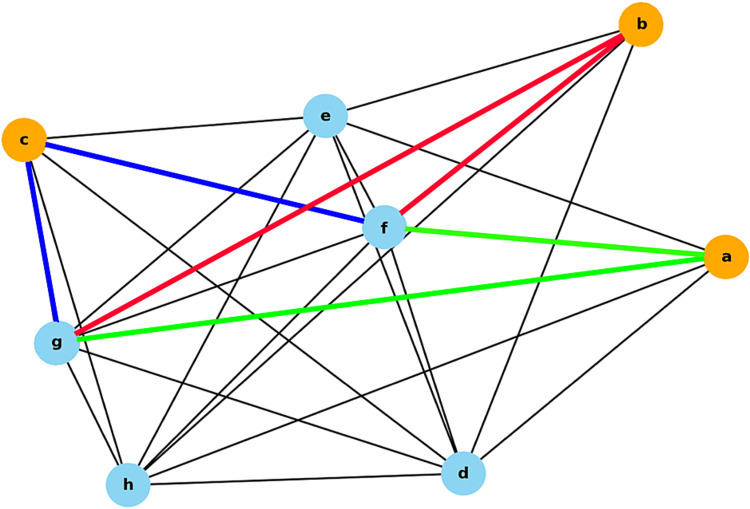
Routes from f to g including one orange node a: TVT, b: TOT, c: VEL, d: AGE, e: PVR, f: Δ1-hrPadTest, g: ΔOABSS, h: ΔICIQ-SF The nodes for the treatment methods are orange. The other variables are indicated in light blue. The lengths of the edges represent the correlation coefficients, as listed in Table 1. TVT: Tension-free Vaginal Tape, TOT: Transobturator Tape, VEL: Vaginal non-ablation Erbium YAG laser, PVR: Post-void Residual, 1-hrPadTest:1-hour Pad Test, OABSS: Overactive Bladder Symptom Score, ICIQSF: International Consultation on Incontinence Questionnaire Short Form. Δ1-hrPadTest: The value obtained by subtracting the post-treatment 1-hrPadTest score from the pre-treatment score, g: ΔOABSS: The value obtained by subtracting the pre-treatment OABSS score from the post-treatment score, h: ΔICIQ-SF: The value obtained by subtracting the pre-treatment ICIQ-SF score from the post-treatment score. Green Line: Route from f to g including a, Red Line: Route from f to g including b, Blue Line: Route from f to g including c.

Using the above code, this function estimated the most effective treatment for “f: Δ1-hrPadTest” and “g: ΔOABSS” based on the given correlation coefficient data. The function calculated the total correlation coefficients associated with each treatment method and selected the one with the highest total value. Figure 2 shows that when applying this code to the current database, there are three paths (blue, red, and green lines) in the network diagram from f to g, each including at least one orange node. Among these, the route including c is the shortest (blue line), which can be visually understood.

## Discussion

The primary objective of this study is to optimize the selection of treatments for urinary incontinence using discrete mathematics, particularly network diagrams and heuristic functions. We hypothesized that these mathematical tools could enhance the decision-making process by providing a more structured visual approach when evaluating treatment options based on individual patient symptoms and treatment history.

The innovative aspect of this study lies in its integration of discrete mathematics into the clinical decision-making process. Traditional UI treatment approaches largely rely on empirical experience and general treatment guidelines [9,10]. However, as our study demonstrated, it provides a new method for visualizing the interrelationships between various treatment options and patient-specific factors. The application of network diagrams enables the visualization of functional relationships between different nodes using a one-dimensional layout that preserves the hierarchical modular organizational information [11]. This visualization not only helps identify the most appropriate strategies but also assists professionals in effectively explaining these choices.

In our approach, we utilized network diagrams to map the relationships between different UI treatments, their potential outcomes, and associated risks. By inputting individual patient data such as specific symptoms or past treatment responses, the diagram dynamically indicates the optimal treatment pathways. This personalized approach began as a study on how to map treatment relevance, potential outcomes, and associated risks onto network diagrams based on individual patient data [11,12].

Heuristic functions played a key role in our study, serving as a tool for estimating the effects and risks associated with each treatment option. Heuristic functions and similar approaches have been suggested to be useful in other medical and research contexts [13]. These functions allow for rapid assessment, enabling healthcare providers to make informed decisions without the need for time-consuming analyses. This aspect is particularly beneficial in clinical settings where time is a critical factor.

Compared with current treatment practices, our methodology offers a more data-driven and personalized approach. Although current practices are effective, they often do not consider the unique circumstances of each patient. It has been reported that TVT may worsen symptoms of OAB in some cases [14]. The polypropylene mesh used in TVT and TOT has been reported to cause pain in some patients and form pathological granulomas [15]. In contrast, laser treatment has been reported to be safe with fewer side effects, and there is evidence of its efficacy in randomized controlled trials) [16,17]. However, even with VEL, the effects last only a few years, necessitating regular treatment [18,19]. Our approach, by considering the history and symptoms of individual patients, has the potential to bridge this gap and enhance the effectiveness of the selected treatment.

These findings have significant implications for future research and clinical practice. In future research, this study will pave the way for further exploration of the use of discrete mathematics in other medical fields. In clinical practice, heuristic decision-making, an efficient cognitive process that ignores some information, can lead to more accurate judgments than using complex strategies, potentially resulting in improved patient outcomes [20].

The next research topic is navigation accuracy when the variables are increased. For example, the data discussed in this study include age and PVR as variables, but it is important to repeat tests with datasets that contain many variables, such as BMI, childbirth experience, number of vaginal deliveries, infertility treatment, hypertension, hypercholesterolemia, blood urea nitrogen, creatinine, and estimated glomerular filtration rate, among others.

Despite these promising results, this study had some limitations. The primary limitation is the reliance on accurate patient data for effective network diagrams and heuristic functions. Inaccurate or incomplete data can lead to suboptimal treatment recommendations. Future research should focus on improving the accuracy and completeness of the patient data collection. Additional studies are required to validate the effectiveness of this approach in a broader patient population.

## Conclusions

This study highlights the potential of discrete mathematics in enhancing decision-making for urinary incontinence treatment, particularly through the use of network diagrams and heuristic functions. This more structured and individualized approach, in line with previous research, indicates that VEL is a treatment for both SUI and UUI. This methodology provides patients with materials that are visually easy to understand within the complex realm of incontinence treatment strategies, potentially leading to improved outcomes. To fully harness the potential of this innovative approach, further research and clinical trials are necessary.
